# Design and validation of a reporter mouse to study the dynamic regulation of TFEB and TFE3 activity through *in vivo* imaging techniques

**DOI:** 10.1080/15548627.2024.2334111

**Published:** 2024-03-24

**Authors:** Electra Brunialti, Nicoletta Rizzi, Rita Pinto-Costa, Alessandro Villa, Alessia Panzeri, Clara Meda, Monica Rebecchi, Donato A. Di Monte, Paolo Ciana

**Affiliations:** a Department of Health Sciences, University of Milan, Milan, Italy; b Animal Care Unit, University of Milan, Milan, Italy; c German Center for Neurodegenerative Diseases (DZNE), Bonn, Germany

**Keywords:** Autophagy, drug discovery, lysosomal pathway, luciferase, non-invasive study of TFEB and TFE3, optical imaging

## Abstract

TFEB and TFE3 belong to the MiT/TFE family of transcription factors that bind identical DNA responsive elements in the regulatory regions of target genes. They are involved in regulating lysosomal biogenesis, function, exocytosis, autophagy, and lipid catabolism. Precise control of TFEB and TFE3 activity is crucial for processes such as senescence, stress response, energy metabolism, and cellular catabolism. Dysregulation of these factors is implicated in various diseases, thus researchers have explored pharmacological approaches to modulate MiT/TFE activity, considering these transcription factors as potential therapeutic targets. However, the physiological complexity of their functions and the lack of suitable *in vivo* tools have limited the development of selective MiT/TFE modulating agents. Here, we have created a reporter-based biosensor, named CLEARoptimized, facilitating the pharmacological profiling of TFEB- and TFE3-mediated transcription. This innovative tool enables the measurement of TFEB and TFE3 activity in living cells and mice through imaging and biochemical techniques. CLEARoptimized consists of a promoter with six coordinated lysosomal expression and regulation motifs identified through an in-depth bioinformatic analysis of the promoters of 128 TFEB-target genes. The biosensor drives the expression of luciferase and tdTomato reporter genes, allowing the quantification of TFEB and TFE3 activity in cells and in animals through optical imaging and biochemical assays. The biosensor’s validity was confirmed by modulating MiT/TFE activity in both cell culture and reporter mice using physiological and pharmacological stimuli. Overall, this study introduces an innovative tool for studying autophagy and lysosomal pathway modulation at various biological levels, from individual cells to the entire organism.

**Abbreviations:** CLEAR: coordinated lysosomal expression and regulation; MAR: matrix attachment regions; MiT: microphthalmia-associated transcription factor; ROI: region of interest; TBS: tris-buffered saline; TF: transcription factor; TFE3: transcription factor binding to IGHM enhancer 3; TFEB: transcription factor EB; TH: tyrosine hydroxylase; TK: thymidine kinase; TSS: transcription start site.

## Introduction

TFEB belongs to the family of microphthalmia-associated transcription factor (MiT/TFE) along with three other evolutionarily conserved members: MITF, TFE3, and TFEC [1]. This family of helix-loop-helix leucine-zipper proteins generate homo- or hetero-dimers, which can bind to the specific DNA responsive elements in the promoter of target genes and transcriptionally regulate their expression.

TFEB recognizes the CACGTG E-box motif, which is also recognized by other transcription factors such as MYC, MAX, and MAD [2–4], and the MiT-specific TCATGTG M-box sequence [5]; the sequences flanking the motifs are able to confer transcription factor specificity [2–5]. TFEB, through direct binding to the coordinated lysosomal expression and regulation (CLEAR) motif [2], coordinates the expression of genes involved in lysosome biogenesis and function, autophagy, lipid catabolism, and oxidative phosphorylation [2–4,6]. The subcellular localization and activity of TFEB are regulated by post-translational modifications and protein-protein interactions. Under high-nutrients conditions, TFEB is phosphorylated by MTOR (mechanistic target of rapamycin kinase) and remains inactive in the cytosol, however, in response to starvation, or lysosomal stress, TFEB is rapidly dephosphorylated and translocates into the nucleus where induces the transcription of its target genes [3,4]. Interestingly, its closely related paralog, TFE3, is also able to bind CLEAR sequence [7] and has been found to regulate a gene pool that is largely superimposed to the one orchestrated by TFEB [7]. Both transcriptions factors reciprocally cooperate during the adaptive response of whole-body metabolism [8] and have at least in part redundant and cooperative functions [8]. The primary distinction among these genes is their differential expression levels in distinct cell types [9].

TFEB and TFE3 play various roles in different organs and tissues. They are involved in coordinating metabolism and cell differentiation in muscle [6,8] and liver [10,11], regulate bone mass in the skeleton [12] and in the immune system play a role in the innate immune response [13] and myeloid cell differentiation [14]. TFEB and TFE3 activation can also contribute to the cellular stress response mechanism [15], as it is induced by lysosomal dysfunction, infection [16], inflammation [8], mitochondrial damage [17], and endoplasmic reticulum stress [18,19].

Dysfunction in TFEB and TFE3 signaling has been observed in neurodegenerative diseases, lysosomal storage disorders, and various types of cancer [15,20]; consistent with this concept, strategies targeting TFEB and TFE3 have shown promise in promoting cellular clearance in cellular and animal models of diseases characterized by the accumulation of metabolic intermediate products or protein aggregates [21], including lysosomal storage diseases [21,22], Parkinson [23–25], Alzheimer [26–28], Huntington diseases [29], SERPINA1/α1-anti-trypsin deficiency [30], spinal bulbar muscular atrophy [31] and diet-induced obesity [11]. While, several lines of evidence suggest that the identification of clinically compatible MiT/TFE inhibitors may offer a rational therapeutic avenue for the treatment of pathologies induced by TFEB, TFE3 and MITF overexpression or hyperactivation, such as MiT-renal cell carcinoma [32], Birt-Hogg-Dubé syndrome [33], tuberous sclerosis [34,35] and malignant melanoma [36]. Despite the large body of preclinical data suggesting a therapeutic potential of modulating MiT/TFE member, there are currently no clinical applications. A variety of reasons may explain this lack of pharmacological/clinical development; in particular, the complexity of TFEB and TFE3’s functions has likely hindered the development of TFEB and TFE3 modulators with sufficient specificity to limit potential side effects [37]. Furthermore, the availability of experimental tools that would permit a detailed and specific assessment of TFEB and TFE3 function and drug modulation is presently quite limited.

Reporter-based biosensors have been successful tools for screening transcription factor modulators [38] enabling the high-throughput screening in cell lines [39] and tissue profiling of drug activity in reporter mice [40–42]; therefore, the generation of reporter systems to study TFEB and TFE3 modulation might fill the present methodological gaps.

In this study, we describe the development and validation of a novel reporter that enables the dynamic measurement of TFEB and TFE3 activity under physiological conditions and in response to pharmacological modulation in cells and mice. This biosensor, based on the reporter, along with imaging, cellular, and biochemical assays, allows for the characterization of TFEB and TFE3 activity across multiple levels, ranging from tissues to individual cells. By more precisely assessing the kinetics and dynamics of changes of these transcription factor activity, this approach will likely represent a valuable new tool for the identification of specific and promising TFEB and TFE3 modulators [43].

## Results

### Generation of a reporter system of TFEB activity: a tool for monitoring the regulation of lysosome biogenesis and autophagy pathways

To develop a reporter system for monitoring the regulation of lysosome biogenesis and autophagy pathways, we conducted a bioinformatics analysis of the promoter regions of 128 TFEB-target genes involved in these pathways (see list in Table S1) [3,6,11]. The goal was to identify common features that confer TFEB specificity to the promoter response. We analyzed the nucleotide composition of the E-box-like sequence recognized by TFEB (CLEAR motif) [2] and the adjacent nucleotides in these selected genes. Additionally, we examined the distance of CLEAR elements from the transcription start site (TSS), the presence of multiple elements, and the distance between them. Our analysis confirmed that CLEAR elements are typically clustered in multiple copies, more frequently located within −200 base pairs from the TSS, consistent with previous reports [2,3]. Based on these important features for efficient responsiveness to TFEB, we designed an “optimized” promoter that was computationally validated using JASPAR2020. We identified putative responsive elements in our synthetic promoter and underwent an iterative process to modify the sequence, reducing the likelihood of undesired binding sites for transcription factors other than TFEB (Figure S1). The resulting synthetic TFEB-responsive sequence, named CLEARoptimized, consisted of a module of six TFEB-binding elements (Figure S1). These TFEB binding sites showed greater homology with the CLEAR motifs present in the promoter regions of *Gba, Cox8b*, and *Ctsc* genes. They were located between −195 to −118 base pairs upstream the TSS. The CLEARoptimized oligonucleotide was chemically synthesized and cloned upstream of the minimal thymidine kinase (*Tk*) promoter, ensuring the appropriate distance from the TSS. To guarantee an equal transcription of the two reporter genes, luciferase (luc2) [44] and tdTomato [45], we inserted them as a single fusion gene separated by the sequence encoding for the T2A self-proteolytic peptide (Figure S1) [46] downstream of the TSS.

### Validation of the reporter system for TFEB

To assess the efficiency of the new CLEARoptimized element in driving TFEB-dependent transcription of the two reporters, we compared it with the CLEAR element from the *Lamp1* gene (referred to as pCLEARLamp1) [47] and a 2000 bp region of the *Tfeb* promoter (referred to as p*Tfeb*promoter) [29], both well-characterized TFEB-responsive sequences (Figure S1B). Computational analysis using the JASPAR2020 software revealed significant differences in TFEB-recognition selectivity between the CLEARLamp1 and CLEARoptimized responsive elements. CLEARLamp1 exhibited putative binding sites for at least 13 different transcription factors, including TFEB, TFEC, and TFE3, three members of the MiT/TFE family [48,49]. Furthermore, seven of the thirteen transcription factors binding to CLEARLamp1 (USF2, MITF, BHLHE41, USF1, SREBF2, SREBF1, and ARNT2) displayed higher binding scores than TFEB (Table S2). In contrast, CLEARoptimized displayed putative binding sites only for TFEB, TFEC, and TFE3, with TFEB having the highest score. This suggests that the activity of the synthetic responsive element may be less affected by off-target pathways.

**Figure 1.**
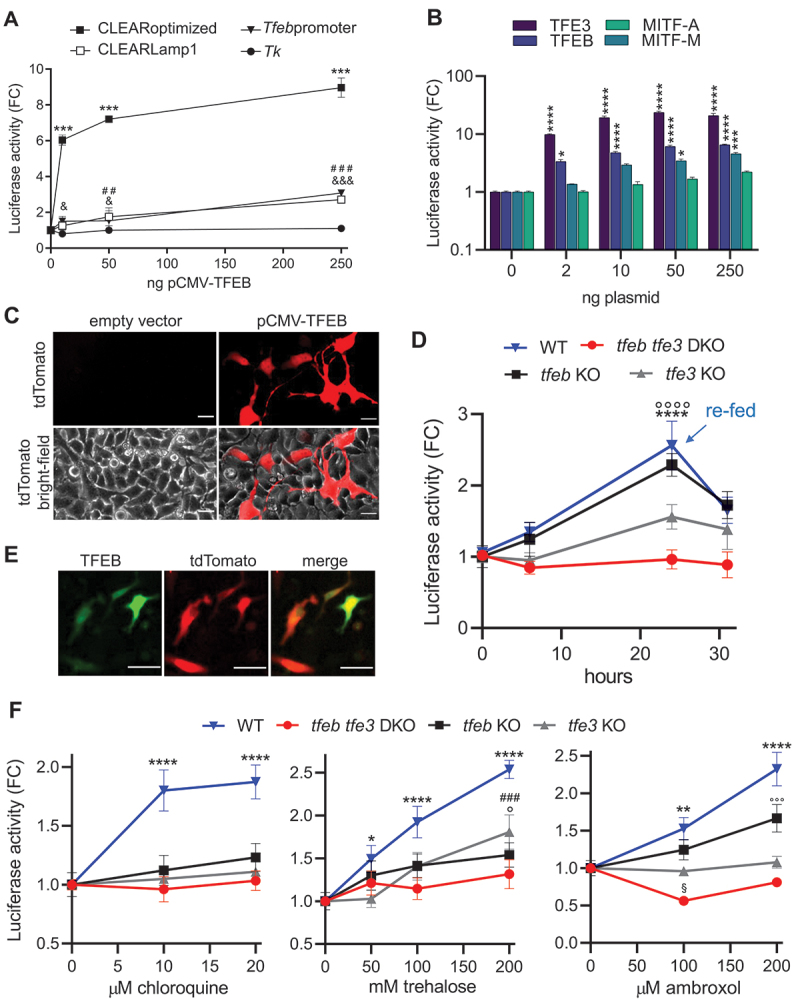
Validation of pCLEARoptimized. (A) Luciferase activity was measured in HeLa cells transiently co-transfected with the indicated reporter vectors and a plasmid expressing the TFEB protein (pCMV-TFEB). Data are mean values ± SD (n = 2) in duplicate, and the luciferase activity is expressed as fold change (FC) of normalized RLU compared to the mean value of the same construct co-transfected with an empty vector. ***p < 0.0001: CLEARoptimized; &<0.05, &&&<0.0001: *Tfeb*promoter; ##<0.005: CLEARLamp1; versus 0 ng pCMV-TFEB calculated by 2-way ANOVA followed by Tukey’s multiple comparisons test. (B) Luciferase activity was measured in RCS cells co-transfected with pCLEARoptimized and increasing concentration of a plasmid expressing TFEB, TFE3, MITF-A or MITF-M. Data represent fold change (FC) of normalized RLU versus vehicle ± SEM (n = 4) in duplicate; *p < 0.05, ***p < 0.0005, ****p < 0.0001 *versus* 0 ng of pCMV-plasmid calculated by 2-way ANOVA followed by Tukey’s multiple comparisons test. (C) Representative pictures of tdTomato fluorescence, and the merge with the bright-field, of the pCLEARoptimized co-transfected with 250 ng of an empty vector or pCMV-TFEB for 24 h. (D) Luciferase activity was measured in RCS cell line transiently transfected with pCLEARoptimized and grown in diluted media. At 24 h, the media was replaced with complete media (re-fed). Data represent FC of normalized RLU versus vehicle ± SD (n = 3) in triplicate; ****p < 0.0001: WT; °°°° *p* < 0.0001: *tfeb* KO *versus* time 0 calculated with one-way ANOVA followed by Dunnett’s multiple comparisons test. (E) Representative pictures of the fluorescence emitted by tdTomato (red) in cells co-transfected with the pCLEARoptimized vector with a vector expressing a fusion protein between TFEB and GFP (TFEB in green), and merge of the two signals. (F) Luciferase activity was measured in RCS cell lines transiently transfected with the pCLEARoptimized and treated with agents capable of eliciting TFEB activation for 16 h: chloroquine, trehalose, ambroxol. Bars represent FC of normalized RLU versus vehicle ± SD (n = 3) in triplicate; *p < 0.05, **p < 0.005, ****p < 0.0001: WT; ^###^*p* <0.001: *tfe3* KO; °*p* < 0.05, °°°p < 0.001: *tfeb* KO; *versus* vehicle calculated with one-way ANOVA followed by Dunnett’s multiple comparisons test.

Next, we compared the three reporter systems (pCLEARoptimized, pT*feb*promoter, pCLEARLamp1) (Figure S1B) carrying different TFEB-responsive elements to determine their ability to report TFEB activity. HeLa cells were transiently transfected with the three constructs along with increasing concentrations of pCMV-TFEB, a vector constitutively expressing TFEB [11] (Figure 1A). These experiments demonstrated that the pCLEARoptimized construct exhibited the greatest and most sensitive response. Luciferase activity in the protein extract increased 6-fold when pCLEARoptimized was co-transfected with a minimal amount of pCMV-TFEB (10 ng), reaching up to 9-fold the basal level with the highest amount of the expression vector. The other reporter systems showed lower sensitivity and magnitude of response. No response was observed when the CLEAR element was deleted from the pCLEARoptimized construct (p*Tk*) (Figure 1A and S1B), indicating that the TFEB-dependent induction could be attributed solely to the synthetic responsive element cloned upstream of the *Tk* promoter. Additionally, fluorescence microscopy analysis of HeLa cells co-transfected with pCLEARoptimized and pCMV-TFEB or an empty vector (as a negative control) showed efficient expression of the tdTomato reporter only in cells overexpressing TFEB (Figure 1C). This relationship was further confirmed using a vector constitutively expressing a TFEB-GFP fusion protein [50], where the red and green fluorescence colocalized (Figure 1E), indicating a TFEB-dependent expression also for the tdTomato. These findings suggest that the CLEARoptimized reporter system could be used to detect TFEB activation at the cellular level. Next, given the abundance of literature data suggesting the striking similarity in regulation and functions among the MiT/TFE family of transcription factors, particularly with TFEB and TFE3 exhibiting partially redundant and cooperative functions [7,10], we assessed the responsiveness of the pCLEARoptimized reporter system to the overexpression of TFE3 and MITF. HeLa cells were transiently co-transfected with constructs constitutively expressing TFEB, TFE3, MITF isoform A, or MITF isoform M [51], along with the pCLEARoptimized reporter system. As depicted in the graph (Figure 1B), the expected response (Figure 1A) was observed with the heterologous expression of TFEB. Notably, the construct expressing TFE3 induced a robust reporter response, with luciferase activity in the protein extract increasing by 9-fold with 2 ng of pCMV-TFE3 and approximately 20-fold at higher concentrations of the plasmid. In contrast, a response was detected only at higher doses of the construct for MITF-M isoform (50–250 ng), and a negligible response was observed with the plasmid expressing the MITF-A isoform. This demonstrates the CLEARoptimized reporter’s capability to accurately reflect the activation of the TFEB and TFE3 members of the MiT/TFE family efficiently binding the CLEAR sequence.

### CLEARoptimized responds to the physiological and pharmacological TFEB and TFE3 activation

To evaluate the responsiveness of CLEARptimized in a more physiological condition than transcription factor overexpression, we assessed luciferase expression following cell starvation. Starvation is a well-known mechanism that induces the nuclear translocation of TFEB and TFE3 and stimulates their transcriptional activity [7,11]. Consistent with this, time-course studies were conducted in rat chondrocytes cells (RCS) [19] transiently transfected with pCLEARoptimized (Figure 1D), demonstrating a significant and gradual increase in luciferase activity upon nutrient depletion from the culture medium (achieved by diluting the medium 10 times with Hanks balanced salt solution). The reporter expression showed a 2.5-fold increase at 24 h and decreased after replacing the depleted medium with a complete (undiluted) culture medium. In contrast, no induction of luciferase activity was observed at the same time points in cells normally fed with a complete medium (Figure S2A); the reporter response in cell starvation conditions was also corroborate using wild-type (WT) human-derived HeLa cells (Figure S2B). Interestingly, the luciferase activity precisely mirrored the expected time-course of TFEB activation previously observed under similar starvation conditions [11].

To confirm the transcription factor specificity of the reporter, a starvation experiment was conducted using RCS cells lacking either TFEB (*tfeb* KO), TFE3 (*tfe3* KO), or both (*tfeb tfe3* DKO) transcription factors (Figure 1D, S2A). Interestingly, the luciferase response in *tfeb* KO cells was comparable to that in the WT, while a distinct reduction in the response was observed in *tfe3* KO cells. This reduction ultimately led to a complete lack of response in the double knockout cells, as the luciferase signal remained consistently similar throughout the entire experiment. These data suggest that in RCS cells the CLEAR-mediated response to starvation is mainly due to TFE3 transcriptional activity.

To test the ability of the pCLEARoptimized construct to respond to pharmacological activation of TFEB, HeLa (WT) and RCS cells (WT, *tfeb* KO, *tfe3* KO, *tfeb tfe3* DKO) transiently transfected with pCLEARoptimized were treated for 16 h with increasing concentrations of various TFEB activators or the corresponding vehicle (water). A concentration-dependent increase in luciferase activity was clearly observed with chloroquine [50], trehalose [52], and ambroxol [53] in WT cells (Figure 1F and S2C), while no response was detected in the *tfeb tfe3* DKO cells. Regarding the single KOs, no activation was detected after chloroquine treatment for both cells line; an intermediate response was detected with trehalose, while for ambroxol, a slight luciferase activation was detected only in the *tfeb* KO. This demonstrates that pCLEARoptimized is an efficient and sensitive reporter system for both TFE3 and TFEB activity as the depletion of both transcription factors is sufficient to abolish the reporter response in all the conditions tested. Moreover, the data obtained with the single KO models also suggest a differential participation of each factor in the transcriptional responses to pharmacological activation.

### Generation of Tfeb and Tfe3 reporter mice

**Figure 2.**
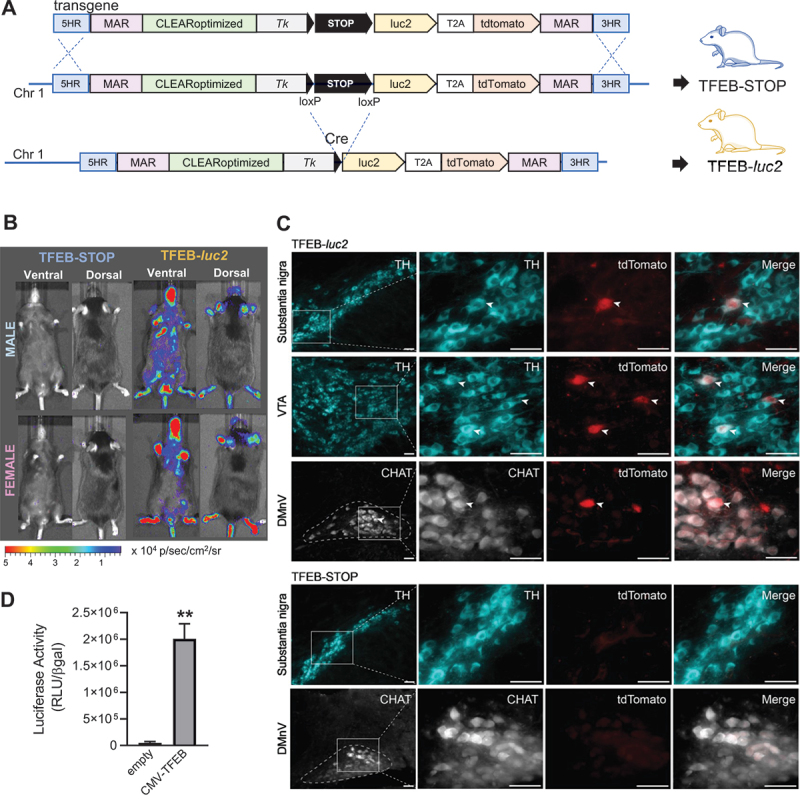
Characterization of TFEB-STOP and TFEB-*luc2* mice. (A) Schematic representation of the transgene inserted in chromosome 1 (Chr1) of reporter mice using homologous recombination prior (upper) to and after excision of the STOP sequence (lower). The reporter mouse with the STOP sequence is called TFEB-STOP. The breeding of this line with B6.C-Tg(CMV-cre)1Cgn/J mice (cre) triggers the excision of the STOP and the generation of the TFEB-*luc2* line. 5 HR, 3 HR: homologous regions for integration into chromosome 1 (Chr1); MAR: matrix attachment regions; CLEARoptimized: TFEB responsive element developed in this work; *Tk*: minimal thymidine kinase promoter; luc2: optimized firefly luciferase 2; T2A: self-proteolytic peptide; tdTomato: tdTomato red fluorescent protein; STOP: POLR2 (RNA polymerase II) termination signal; loxP: locus of X-over P1. (B) Representative pictures of ventral and dorsal luciferase emission of TFEB-STOP and TFEB-*luc*2 mice (female and male). Pseudocolor images of each mouse were obtained 15 min after the subcutaneous injection of 80 mg/kg of luciferin with a 5-min exposure time and reported with corresponding scale bars. (C) Expression of the tdTomato reporter in specific cell types of TFEB-*luc*2 mice. Coronal brain sections of the midbrain and medulla oblongata of TFEB-*luc*2 and TFEB-STOP mice were double-stained with a tdTomato antibody together with an antibody against TH (tyrosine hydroxylase; midbrain) or CHAT (choline acetyltransferase; medulla oblongata). Squares in the lower magnification images (panels on the left) encompass areas of the substantia nigra pars compacta, ventral tegmental area (VTA) and dorsal motor nucleus of the vagus nerve (DMnV; delineated by dashed lines) that are enlarged on the right. Co-immunoreactive neurons (arrowheads) were observed in sections from TFEB-*luc*2 but not TFEB-STOP mice. Scale bars: 50 μm. (D) Luciferase activity in lung homogenate dissected 24 h after the *in vivo* transfection of TFEB-*luc2* mice with an empty vector (empty) or a vector allowing the heterologous expression of TFEB (pCMV-TFEB). Bars represent RLU normalized for transfection efficiency ± SD (*n* = 2), **p < 0.01 *versus* empty vector calculated with unpaired t-test.

Given the high specificity and sensitivity of the CLEARoptimized reporter system for TFEB, we decided to utilize this system to generate a Tfeb and Tfe3 reporter mouse for studying the modulation of the activity of these transcription factors *in vivo*. To achieve this, we employed a well-established technology in our laboratory for generating reporter mice. This technology involves: i) inserting the transgene into a specific locus on chromosome 1 of the mouse genome, which has been previously characterized as a transcriptionally active locus for transgene expression [54], and ii) flanking the reporter system with insulator sequences to prevent any position effects [55] (Figure 2A). Indeed, the insertion of a transgene flanked by insulator sequences in an actively transcribed locus has been shown to be an effective strategy for minimizing the influence of surrounding chromatin on transgene expression, thereby ensuring the generation of reliable transgenic reporter mice [40,42,51,54,56].

Based on these considerations, we constructed a knock-in vector containing the CLEARoptimized reporter system flanked by matrix attachment region (MAR) sequences from the chicken lysozyme gene (Figure 2A). Additionally, the reporter system was modified to include a floxed STOP sequence (POLR2 [RNA polymerase II] termination signal) [54] between the promoter and the reporter cassette (Figure 2A). This modification allows for tissue-specific expression of the reporter system simply by crossing the reporter mouse with a transgenic mouse expressing Cre recombinase in a tissue-specific manner [54]. The knock-in procedure was successfully performed, resulting in the generation of a clone that displayed the expected band pattern upon diagnostic PCR, indicating homologous recombination of the full transgenic cassette with the mouse genome (Figure S3A). The amplified fragments were also sequenced to confirm correct insertion. Injecting this clone into mouse blastocysts enabled the generation of a reporter mouse line with the entire transgene (including the STOP sequence) inserted into the genome, which we named TFEB-STOP (Figure 2A). As anticipated, TFEB-STOP mice did not exhibit detectable luciferase activity when subjected to *in vivo* and *ex vivo* imaging acquisitions (Figure 2B, Figure S3B and Figure S3C). However, when the TFEB-STOP line was crossed with the B6.C-Tg(CMV-cre)1Cgn/J mouse, which expresses Cre recombinase in germ cells [57], the floxed STOP sequence was excised from the genome (Figure 2A), an alteration that occurs in the germ line genome and is transmitted to the offspring regardless of the presence of the Cre enzyme. The derived pups, resulting in the generation of the TFEB-*luc*2 line, were then crossed with C57BL/6 for nine generations to remove the Cre transgene and obtain a reporter line in a full C57BL/6 background (Figure 2A).

### Reporter expression in TFEB*-luc2* mice

Two sets of analyses were carried out in TFEB-*luc2* mice to verify that germline removal of the STOP sequence correctly released the transcriptional inhibition of the reporter system. First, basal expression of luciferase was assessed in male and female TFEB-*luc2* mice. Results showed bioluminescence that was emitted from the whole body of these animals and remained relatively stable throughout adulthood (Figure 2B and S3D). Bioluminescence imaging of luciferase activity is a versatile assay but lacks sufficient spatial resolution for detection of the reporter system within tissues and specific cell types. This limitation would be overcome in our TFEB-*luc*2 model by the CLEAR-regulated expression of tdTomato. The second set of analyses was therefore aimed at demonstrating tdTomato expression in TFEB-*luc*2 as compared to TFEB-STOP animals. Immunohistochemistry was carried out in a tissue, namely brain tissue, characterized by anatomically distinct regions and cell populations with specific phenotypes. When tissue sections were stained with anti-tdTomato, robustly labeled cell bodies were observed throughout the brain of TFEB-*luc*2 but not TFEB-STOP mice (Figure 2C and S4). Double-immunolabeling was then carried out to detect tdTomato expression within specific neuronal populations, in particular dopaminergic neurons in the substantia nigra pars compacta and cholinergic neurons in the dorsal motor nucleus of the vagus nerve (DMnV). Colabeling of nigral neurons with anti-tdTomato and anti-tyrosine hydroxylase (TH) revealed colocalization of the reporter protein and the dopaminergic cell marker in tissue sections from TFEB-*luc*2 mice; quite in contrast, samples from TFEB-STOP animals showed TH immunoreactive cells that were consistently devoid of tdTomato expression (Figure 2C). Similarly, staining of medullary DMnV neurons with antibodies against tdTomato and choline acetyltransferase (a cholinergic cell marker) revealed colabeled neurons only in TFEB-*luc*2 mice (Figure 2C). Taken together, these results demonstrated clear expression of both luciferase and tdTomato in our reporter mice and supported the suitability of this model to assess reporter expression at whole body, organ and tissue levels and with cellular resolution.

### TFEB*-luc2* reporter mouse validation

**Figure 3.**
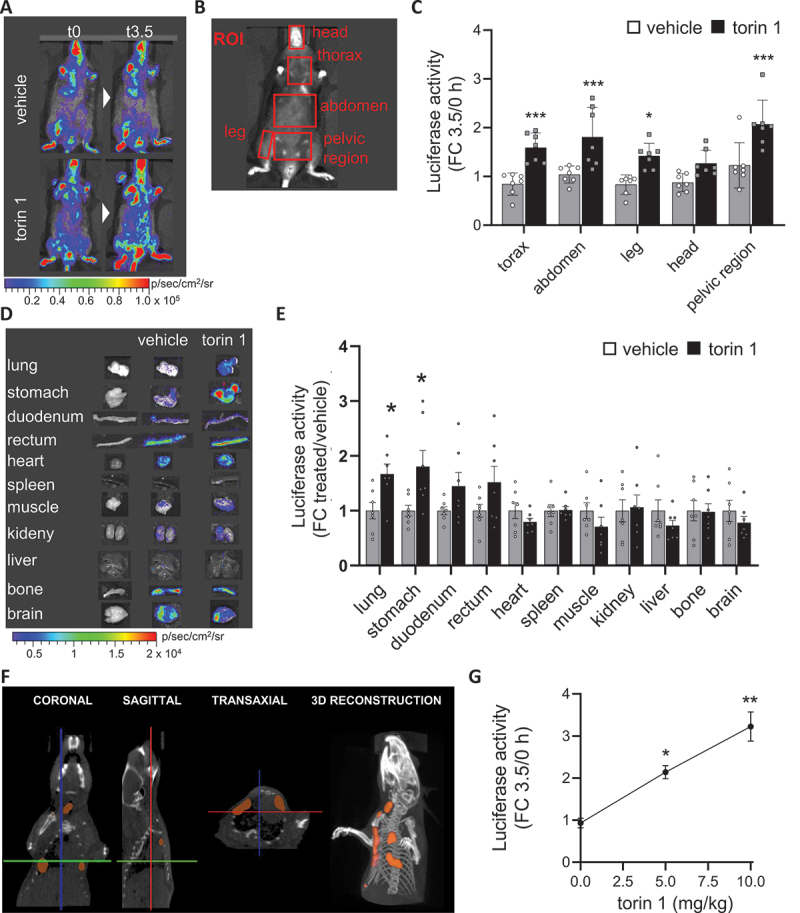
TFEB*-luc2* reporter mouse responds to pharmacological TFEB activation. (A) Representative *in vivo* imaging of TFEB-*luc2* mice treated with torin 1 (5 mg/kg) or vehicle at 0 or 3.5 h after the treatment. Pseudocolor images of bioluminescence are represented according to the reported scale bar. (B) *In vivo* bioluminescence was quantified in selected regions of interest (ROIs: red square). (C) Quantification of the bioluminescent light from ROIs of mice treated as in A. Bars represent photon emissions (p/s/cm^2^/sr) expressed as fold induction versus time 0 ± SD (*n* = 7); *p < 0.05, ***p < 0.001 *versus* vehicle calculated with one-way ANOVA followed by Sidak’s multiple comparisons test. (D) Pseudocolor image of the bioluminescence emitted from representative organs dissected 3.5 h after torin 1 or vehicle treatment and (E) bioluminescence quantification. Bars represent photon emissions (p/s/cm^2^/sr) expressed as fold induction versus vehicle ± SD (*n* = 7); *p < 0.05, *versus* vehicle calculated with one-way ANOVA followed by Sidak’s multiple comparisons test. (F) Coronal, sagittal and, transaxial section and 3D reconstruction of the X-ray and bioluminescence signal derived from the thoracic area of a TFEB-*luc2* mouse injected with 5 mg/kg of torin 1; the area of the bioluminescent signal is represented in orange. (G) Quantification of the bioluminescent light from the whole body of mice treated with 0, 5, 10 mg/kg of torin 1. Y axes represent photon emissions (p/s/cm^2^/sr) expressed as fold induction versus time 0 ± SD (*n* = 2); *p < 0.05, **p < 0.005 *versus* vehicle calculated with one-way ANOVA followed by Dunnett’s multiple comparisons test.

To demonstrate the expression of reporter genes in TFEB-*luc2* mice as a result of TFEB activation, the reporter mouse was subjected to various stimuli known to activate the transcription factor. These stimuli included TFEB overexpression and administration of torin 1, an MTOR inhibitor that promotes TFEB nuclear accumulation (Figure S2D) and transcriptional activity [58]. TFEB overexpression in specific mouse tissues was achieved through *in vivo* transient transfection. For this purpose, the pCMV-TFEB construct or an empty vector as a negative control were encapsulated into lipid nanoparticle formulations [59] and administered intravenously via retro-orbital veins to the reporter mouse. Previous reports have shown that 24 h after administration, a peak of plasmid accumulation can be found in the lung [59]. This tropism for the lung was also demonstrated in our preliminary experiments, where the nanoparticles were loaded with a plasmid (pCMV-luc2) constitutively overexpressing the *luc2* reporter gene and administered intravenously to wild-type mice (Figure S5A). When pCMV-TFEB or the empty vector were encapsulated in the nanoparticles and administered intravenously to the TFEB-*luc2* line, the quantification of luciferase in the lung 24 h after injection confirmed a specific upregulation of reporter expression by approximately 40 times compared to the negative control (Figure 2D), which is reflecting the difference in TFEB expression observed between the two experimental groups (Figure S5B). This demonstrates that the reporter mice are capable of sensing and reporting the activation of the TFEB pathway due to the overexpression of the transcription factor. Next, we investigated whether luciferase was induced in the tissues of TFEB-*luc2* mice by administering torin 1. Previous reports have shown that an intraperitoneal injection of 20 mg/kg of torin 1 is sufficient to suppresses MTORC1 activity in lung from 2 up to 6 h [60]. Two groups of seven mice each were intraperitoneally injected with 5 mg/kg of torin 1, or vehicle and the luciferase activity was analyzed 3.5 h after the treatment (Figure 3A). The dose of 5 mg/kg was chosen to reduce any possible toxic effects that can be induced by the dosage of 20 mg/kg.

During *in vivo* imaging acquisitions, bioluminescence was recorded in different regions of interest (ROIs) (Figure 3B), and we observed an increased photon emission in the thorax, abdomen, leg, and pelvic areas, while a lesser increase in emission was observed in the head (Figure 3C). The profile of luciferase expression in the internal organs was measured by *ex vivo* imaging, demonstrating a clear increase in the bioluminescent signal in the lung and stomach, and a trend toward an increase in the intestine compared to the vehicle-treated mice. However, the signal remained stable in the heart, spleen, muscle (gastrocnemius), kidney, liver, bone, and brain (Figure 3D,E). This is consistent with the expected profile of torin 1 activity after intraperitoneal administration, where MTORC inhibition is expected to be particularly pronounced in the lung [60] and as verified with western blot analysis on lung homogenate (Figure 3E and S5C).

To assess the usefulness of TFEB-*luc2* mice for 3D optical imaging, a microCT scan with DLIT 3D reconstruction was performed on the thoracic area of a TFEB-*luc2* mouse injected with 5 mg/kg of torin 1. As shown in Figure 3F and supplementary video 1, the area of bioluminescence superimposed with the lungs, consistent with the *ex vivo* imaging results (Figure 3D).

Finally, three groups of two mice each were intraperitoneally injected with 5 or 10 mg/kg of torin 1 or vehicle. The quantification of bioluminescence emitted from the whole body (Figure 3G) allowed us to identify a dose-response activation of the reporter, suggesting that the biosensor can be pharmacologically activated in a dose-response manner.

In conclusion, the analysis of luciferase activity in the TFEB-*luc2* reporter mouse enables the profiling of drug action in the whole organism.

### TFEB-*luc2* reporter mouse respond to food deprivation

**Figure 4.**
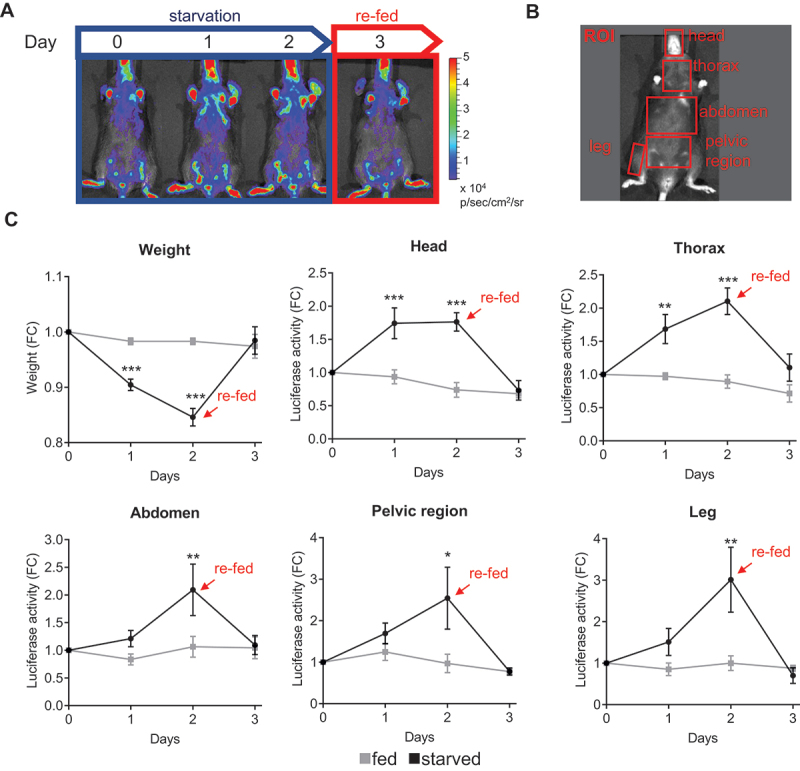
TFEB-*luc2* reporter mouse responds to starvation. (A) Schematic representation of the experiments: TFEB-*luc2* mice were subjected to starvation for 48 h, and then the food was reintroduced for 24 h. The bioluminescence signal is represented using pseudocolors according to the provided scale bar. (B) *In vivo* bioluminescence was quantified in selected regions of interest (ROI, red squares). (C) The weight of the mice was recorded during the experiment. Data represent the weight (g) expressed as fold induction (FC) relative to time 0. Bioluminescence imaging quantifications of the photon emission from the ROIs shown in B are reported in the respective graphs. The measurements of bioluminescence signal (luciferase activity) are presented in the graph as fold change (FC) of the radiance photons measured at different time points *versus* the radiance photons measured at time 0. Data are presented as mean ± SEM (*n* = 9). *p < 0.05, **p < 0.01, ***p < 0.001 versus fed animals calculated with one-way ANOVA followed by Sidak’s multiple comparisons test.

**Figure 5.**
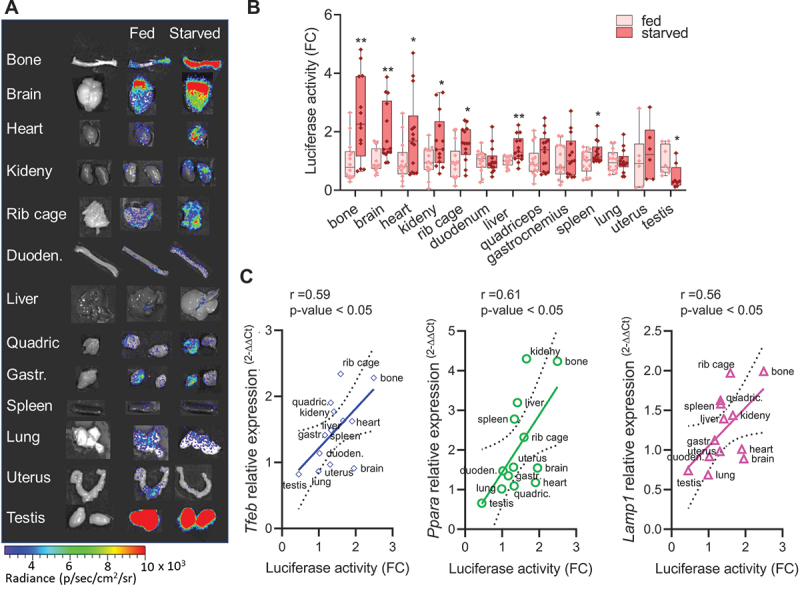
Luciferase activity correlates with TFEB target genes expression. (A) Representative *ex vivo* bioluminescence imaging of the organs dissected from fed or starved mice. Bioluminescence signals were acquired for each organ obtained from mice subjected to 48 h of starvation or normal feeding and are shown as radiance photons (p/s/cm^2^/sr) represented with pseudocolors according to the reported scale bar. Quantification of the bioluminescence signals from the organs is reported in (B). The measurements of bioluminescence signal are presented in the graph as fold change (FC) of the radiance photons of starved *versus* fed animals and presented as mean ± SD of *n* = 7 independent samples measured in duplicate (6 females, 8 males). Statistical significance was determined by one-way ANOVA followed by Sidak’s multiple comparison test versus fed animals. *p < 0.05, **p < 0.01. (C) Total RNA was purified from dissected organs (B), and the expression of TFEB target gene mRNA (*Tfeb*, *Ppara*, *Lamp1*) was analyzed by real-time PCR. Relative quantification of the transcript was obtained using the 2^−ΔΔCt^ method versus the fed samples and was correlated to the luciferase activity for each organ. Pearson r and p-values for each interpolated curve are reported.

As also previously stated, TFEB is induced by food deprivation, which induces its nuclear translocation and increases its levels through an autoregulatory feedback loop [11]. Therefore, we have investigated if the TFEB-*luc2* model responds to this physiological stimulation by increasing the expression of luciferase. To this aim, we performed a series of *in vivo* imaging acquisitions during starvation in the same group of mice to assess the bioluminescent emission (Figure 4A). Two groups of nine TFEB-*luc2* mice each were treated under identical conditions, except that the food was removed for the starved group (time 0). *In vivo* imaging sessions were conducted at 0, 24, and 48 h after food removal, as well as 24 h after food reintroduction (Figure 4A). To serve as a starvation control, we also measured the mouse weight at the same time points (Figure 4C). We quantified the bioluminescence emitted from the ROIs (Figure 4B) and results clearly demonstrate that photon emission increased over time in all the ROIs (head, thorax, abdomen, pelvic region, leg), in parallel with the decrease in body weight (Figure 4C). The signal reached its maximum activity at 48 h and returned to the basal level 24 h after food reintroduction (Figure 4C). In contrast, the luciferase signal remained stable in fed animals, suggesting that food deprivation directly modulates luciferase expression. We further quantified luciferase activity in protein extracts from dissected organs at 48 h after food removal (the time when the signal peaked). The analysis revealed a statistically significant increase in luciferase expression in bone (both the bone and bone marrow components, as shown in Figure S5D), brain, heart, kidney, rib cage, liver, and spleen. A trend toward increased luciferase activity was observed in quadriceps, gastrocnemius, and uterus, while no changes were detected in the duodenum and lung. A reduction in luciferase activity was measured in the testis (Figure 5A,B). Importantly, luciferase expression correlated well with a statistically significant increase in the mRNA expression of TFEB target genes, including *Tfeb* itself, *Ppara*, and *Lamp1*, as measured in the explanted organs (Figure 5C).

Taken together, these results demonstrate that in TFEB-*luc2* reporter mice, *in vivo* and *ex vivo* imaging provide a comprehensive spatial and temporal profile of Tfeb activation in the mouse body.

## Discussion

The molecular and physiological complexity of the TFEB and TFE3 pathways currently hinders the development of drugs that selectively target these transcription factors to produce a specific desired response while minimizing unwanted effects [61]. The overlapping pattern of gene expression shared with other families of transcription factors and the resulting modulation of genes involved in various stress pathways partly explains the diverse therapeutic effects achieved by activating or inhibiting MiT/TFE factors. TFEB or TFE3 activation has been shown to be beneficial for improving neuronal health [62], insulin sensitivity [6], and cardiomyocyte survival during ischemia-reperfusion damage in the heart [63]. On the contrary, inhibiting TFEB and TFE3 activity has demonstrated beneficial effects by reducing cancer cell viability and increasing their susceptibility to anticancer therapies [31,64–66]. Therefore, dissecting the specific contribution of TFEB and TFE3 modulation in physiological and pathological conditions should be a prerequisite for identifying therapeutically useful molecules that selectively activate or inhibit the transcription factors in specific tissues. However, to reach this aim, current methodologies require analyzing a large panel of targets or utilizing transcriptomic analyses, both of which are expensive, time-consuming, and not easily applicable to spatial-temporal investigations of the TFEB and TFE3 pathway during pathogenetic processes or after pharmacological treatments.

In our study, we have developed reporter cells and mice where it is possible to measure MiT/TFE members transcriptional regulation *in vitro* and *in vivo* with simple and direct measures based on biochemical or multimodality imaging assays. We achieved great selectivity of the biosensor for TFEB and TFE3 activity through extensive bioinformatic analysis on the promoters of genes belonging to the CLEAR network [3,6,11]. The selected CLEARoptimized sequences was able to detect the overexpression of the MiT/TFE members and demonstrated unprecedented responsiveness of TFEB/TFE3 to pharmacological and physiological stimulations compared to previously published responsive elements. The cellular experiments confirm that the reporter is a valuable tool for studying the modulation of MiT/TFE factors, which bind to CLEAR sequences to activate their response. The potential application of the reporter system to decipher the mechanisms that drive cellular responses to defined stimuli could be achieved by utilizing the CLEARoptimized reporter system in conjunction with KO cells for specific MiT/TFE members. Our experiments highlight that stimuli known to activate TFEB and TFE3 (such as starvation, trehalose, chloroquine, and ambroxol) trigger distinct, transcription factor-specific cellular responses. Therefore, this strategy may significantly contribute to easily characterizing the mechanism of action of candidate drugs that regulate TFEB andTFE3.

When integrated in the chromatin context, the reliability of the reporter system was ensured by flanking the reporter constructs with insulators preventing positional effects and using a knock-in approach to target the biosensor in a constitutively open region of the chromatin [54,56,67]. Finally, the presence of loxP sequences flanking the transcriptional STOP signal placed downstream the TSS generates a conditional reporter mouse, which may support tissue- or stage-specific detection of TFEB and TFE3 activity for more refined experimental plans. Overall, the generated tool ensures a reliable surrogate marker that selectively reports the TFEB and TFE3 state of activity in cells and mice. The choice of tdTomato and firefly luciferase in the reporter system was instrumental for assessing activation of the MiT/TFE related pathway through a multiplicity of assays at different levels of resolution, from cells to organism level, with qualitative and semiquantitative measurements based on photon emissions or classical biochemical tests, including immunofluorescence and enzymatic assays. The luciferase/luciferin bioluminescent emission was primarily intended for *in vivo* imaging because it is easily detected and quantified with CCD camera-based instruments. This allows for longitudinal and punctual noninvasive identification of transcription factors activation, possibly revealing the anatomical regions in which the activation occurs thanks to the three-dimensional reconstruction of the signal source offered by the latest imaging systems [68]. Additionally, tdTomato fluorescence was intended to allow the analysis of TFEB and TFE3 activity at the cellular level, in living cells, using microscopic techniques, including time-lapse confocal microscopy to study in detail the kinetics of TFEB and TFEB activation following specific treatments. Moreover, the two reporters have different stability in cells and tissues: luciferase has a short half-life, thus may be instrumental for measuring the fast changes occurring in TFEB and TFE3 regulation, while the longer stability of tdTomato may mark all cells and tissues where this regulation has occurred in a more comprehensive picture [44,69].

The experimental validation of our reporter tools provides examples of possible applications in the study of TFEB and TFE3 modulation. The physiological modulation previously observed during starvation [3] was indeed revealed by our biosensors in living cells and animals, allowing a dynamic measure of the activation of the TFEB pathway and the profiling of tissues that display a greater response to starvation. Time course and profiling of the responses are certainly fundamental information for drug development since they enable the identification of the correct time and tissues that a drug should target to obtain an efficacious modulation of TFEB in a pathophysiological condition. Once these parameters are identified, the dynamic profile of activation using a specific molecule, such as torin 1, for example, can be obtained by *in vivo* imaging followed by *ex vivo* imaging analysis of reporter activation in the different organs.

This analysis can be further deepened, providing insights into the cellular complexity characterizing a tissue. As an example of this complexity, we have chosen to analyze the tdTomato reporter in different brain areas where cellular complexity is the most relevant obstacle to the development of functional drugs acting through a ubiquitous target like TFEB, which is involved in a number of homeostatic processes in different areas of the brain [70]. The brain is one of the most intricate organ in the body, with different areas performing different tasks according to area- and cell-specific processes and the mutual connections of its constituent parts [71]. The pathological outcome of neurodegenerative diseases varies depending on which brain region is impacted, and symptoms of these diseases often mirror the physiological function typically performed by the region in question. Due in part to their unique physiology, some regions of the brain and the cells that make up those regions are more vulnerable to disease than others, for instance, to the accumulation of undegraded substrates due to a defect in the autophagy-lysosomal pathways. This highlights the fact that studying brain pathology requires a dissection that goes from the whole brain down to the level of individual cells. By analyzing tdTomato expression in the brain it is possible to pinpoint easily regions of the brain or cells that exhibit high or low levels of the CLEAR-dependent transcriptional activity.

It is well established that the translocation, duplication, and hyperactivation of MiT/TFE members can induce cancer and promote its progression [9]. The TFEB-*luc*2 reporter mouse provides a valuable means to dynamically monitor the CLEAR-dependent transcriptional *in vivo* and dissect of organ-specific and cellular responses, enabling researchers to delve into the intricate mechanisms underlying cancer development. The use of reporters further opens avenues for identifying potential therapeutic targets: high-throughput cellular screening can be applied to select candidate drugs capable of modulating MiT/TFE activity. Additionally, studies related to drug pharmacodynamics and pharmacokinetics can be easily performed using the reporter mouse, facilitating a rapid and cost-effective screening process. This expedites the prioritization of new candidate molecules for different pathology ultimately advancing the field of targeted therapeutics.

Overall, this high-resolution dynamic mapping of TFEB and TFE3 activity allowed by cell and reporter mice is definitely a precious tool for drug screening, as it makes it possible to distinguish the physio-pathological regulation of MiT/TFE or a drug’s action in specific areas and cells, thus allowing testing of the efficacy of a therapeutic strategy in a fast and straightforward way. The application of our reporter system to disease models should considerably improve understanding of the function of TFEB and TFE3 in disease progression and allow screening of medicines selectively targeting this transcription factor.

## Materials and methods

### Plasmids and plasmids construction

The plasmid CLEARoptimized was generated starting from the vector ARE-loxP-STOP1×-loxP-luc2-ires-tdTomato [54] by substituting the following components: 1) the ires sequence with the T2A sequence derived from the pCS2-TAG plasmid (a gift from Shankar Srinivas; Addgene 26,772, RRID:Addgene_26772) [46], using *SnaBI* and *XhoI* restriction sites; and 2) the ARE promoter with the CLARoptimized promoter (synthesized by Eurofins Genomics) using *SacI* and *BssHII* sites. Before the cellular assay, the floxed STOP was excised with the Cre enzyme (New England Biolabs, M0298S) using the standard protocol.

To generate the targeting vector, the CLEARoptimized cassette was cloned into the targeting vector using site-directed recombination (VectorBuilder). In the final construct, the transgene was flanked by MARs and homologous regions for the chromosome 1 locus 19 [54].

The plasmid pCLEARLamp1 (Addgene 66,800; 4XCLEAR-luciferase reporter, RRID:Addgene_66800) [47] and the plasmid p*Tfeb*Promoter (Addgene 66,801; TFEB promoter-luciferase reporter, RRID:Addgene_66801) [47] were gifts from Albert La Spada. The plasmid pCMV-TFEB [3], which expresses constitutively human TFEB protein, was kindly provided by A. Ballabio (Telethon Institute of Genetics and Medicine). The plasmids pEGFP-N1-TFEB (Addgene 38,119; RRID:Addgene_38119), pEGFP-N1-TFE3 (Addgene 38,120; RRID:Addgene_38120), pEGFP-N1-MITF-M (Addgene 38,131; RRID:Addgene_38131), pEGFP-N1-MITF-A (Addgene 38,132; RRID:Addgene_38132), which express fusion proteins of transcription factors (TFEB, TFE3, MITF-M or MITF-A) and GFP, were a gift from Shawn Roczniak-Ferguson [50].

### Generation *of* TFEB-STOP and TFEB*-luc2* reporter mouse

The targeting vector was linearized with *NotI* and transferred into sv6.4 embryonic stem cells by electroporation, using 35 μg of DNA for each 15 million cells (Core Facility for Conditional Mutagenesis, DIBIT San Raffaele). Positive clones were selected with puromycin (1 μg/mL), and one hundred resistant clones were screened for homologous recombination by PCR. A positive clone was injected into C57BL/6NCrL blastocysts and transferred to pseudo-pregnant CD1 females. The resulting chimeric male mice, with approximately 80–90% chimerism, were bred with wild-type C57BL/6J female mice to produce F1 transgenic mice named TFEB-STOP. This line was then crossed with B6.C-Tg(CMV-cre)1Cgn/J mice22 to obtain TFEB-*luc2* reporter mice.

### Cell cultures and transient transfections

HeLa cell lines were obtained from the American Type Culture Collection (CCL-2) and cultured in MEM (Gibco 32,430–027) supplemented with 10% fetal bovine serum (FBS, Euroclone, ECS0186L), 1% streptomycin-penicillin (Gibco 15,240–062), and 1% GlutaMAX (Gibco 35,050–061); WT, *tfeb* KO, *tfe3* KO, and *tfeb tfe3* DKO [19] were kindly provided by Prof. A. Ballabio and Prof. C. Settembre (Telethon Institute of Genetics and Medicine) and cultured in DMEM (Gibco 32,430–027) supplemented with 10% FBS, 1% streptomycin-penicillin, and 1% GlutaMAX. All cell lines were maintained in a humidified atmosphere of 5% CO_2_ and 95% air at 37°C. For transfection 50,000 HeLa or 30,000 RCS cells were seeded in a 24-well plate and cultured overnight prior to transfection. Transfection of HeLa cells were performed using Lipofectamine LTX and PLUS reagent (Thermo Fisher Scientific 15,338) with a DNA:Lipofectamine LTX:PLUS reagent ratio of 0.5:3:0.3 (µg:µL:µL) for each well; transfection of RCS cells were performed using Lipofectamine 3000 reagent (Thermo Fisher Scientific, L3000001) with a DNA:Lipofectamine3000:P3000 reagents ratio of 0.5:0.75:1 (µg:µL:µL) for each well, following the manufacturer’s instructions. Transfected cell lines were treated with ambroxol hydrochloride (Merck, A9797-5 G), chloroquine (Merck, C6628-25 G), and D-(+)-Trehalose (Merck 90,210) dissolved in water for 16 h. For the starvation experiment, the diluted media consisted of 10% complete media diluted in Hanks’ balanced salt solution (Thermo Fisher Scientific 14,025,050). Fluorescence images of live transiently transfected cells were acquired using an Axiovert 200 M microscope with dedicated software (AxioVision Rel 4.9, Zeiss) at a magnification of × 20.

### Animal treatments

All animal experimentation was carried out in accordance with the Animal Research: Reporting of In Vivo Experiments (ARRIVE) guidelines and the European Guidelines for Animal Care. The animal study protocol was approved by the Italian Ministry of Research and University (permission numbers: 5247B.N.459/2019, 445/2018 and 712/2021). The animals were provided ad libitum access to food and housed in individually ventilated plastic cages at a temperature range of 22–25°C with a relative humidity of 50% ± 10%. The housing environment followed an automatic cycle of 12 h of light and 12 h of darkness. To minimize any circadian influence, both the treated and control groups were analyzed at the same time, and the reference point (t0) for the start of the experiments was set in the morning (between 9:00 and 10:00 a.m). For non-viral *in vivo* transfection, in vivo-jetPEI reagent (Polyplus 101,000,040) was used to deliver plasmids into the lungs of 9-week-old mice. The mice were transfected following the standard protocol. In brief, for a 30 g mouse, a mixture of 6.4 µL of in vivo-jet PEI reagent and 40 µg of endotoxin-free plasmid in a 10% glucose solution was injected into the retroorbital vein. The expression was analyzed 24 h later and normalized on the expression of a constitutive plasmid. For the starvation experiment, female and male mice aged 17–25 weeks were placed into clean cages with free access to water and subjected to scheduled *in vivo* imaging sessions. For pharmacological *in vivo* activation, an intraperitoneal injection of 5 mg/kg torin 1 (Callbiochem 475,991) was performed in a saline-based solution containing 20% DMSO (v:v) (Thermo Fisher Scientific, D12345). The luciferase expression was analyzed after 3.5 h.

### *In vivo* and *ex vivo* imaging

For semi-quantitative analysis of photon emission, we followed the standard procedure [72]. Animals were subcutaneously injected with 80 mg/kg of luciferin (Promega, P1041) 15 min before the imaging session. The mice were anesthetized using isoflurane and remained under anesthesia during each 5-minute optical imaging session. Imaging was conducted using a charge-coupled device (CCD) camera (IVIS Lumina II Quantitative Fluorescent and Bioluminescent Imaging, or IVIS SpectrumCT PerkinElmer). After the final *in vivo* acquisition, the mice were euthanized by cervical dislocation. The organs were immediately subjected to a 5-minute *ex vivo* imaging session, followed by fast freezing at −80°C for subsequent assays. Photon emission in different areas was measured using Living Image Software v. 4.2 (PerkinElmer).

### Luciferase enzymatic assay

Luciferase assays were performed as previously described [54]. Briefly, for the cellular assay, the cells were lysed with 1X luciferase cell culture lysis reagent (Promega, E1531) prior to the biochemical assay. For tissues, half of the organs were homogenized in lysis buffer and subjected to freezing and thawing cycles. The protein portion was recovered after centrifugation at 13,000 g for 30 min. The protein concentration was determined using a Bradford assay, and the biochemical luciferase activity assay was carried out in luciferase assay buffer. The relative luminescence units (RLU) were measured using the Veritas luminometer (Turner Biosystems) in a 96-well plate. The RLU determined during a 10-second measurement was expressed as RLU normalized to the protein content measured using the standard Bradford assay.

### Real-time PCR (RT-PCR)

Half of the tissue was dissolved using Trizol reagent (Thermo Fisher Scientific 15,596,026) and subjected to mechanical dissociation. The mixture was then centrifuged at 3,700 g for 10 min at 4°C, and the total RNA was extracted from the supernatant using the Direct-zol RNA Miniprep Kit (Zymo Research, R2050) following the standard protocol. RT-PCR analyses were performed as previously described [72]. Briefly, cDNA synthesis was performed using Moloney murine leukemia virus reverse transcriptase (Promega, M3681) and random primers (Promega, C118A). For each sample, control reactions were performed without the addition of reverse transcriptase. RT-PCR was carried out using SYBR Green chemistry (Promega, A600150), and cDNA was amplified in triplicate in a 96-well plate using GoTaq qPCR Master Mix technology (Promega, A6001) according to the manufacturer’s protocol, in a final volume of 10 μL using a QuantStudio 3–96-Well 0.1 mL Block (Thermo Fisher Scientific). The following thermal profile was used: 2 min at 95°C, followed by 40 cycles of 15 s at 95°C and 1 min at 60°C. The primers used are listed in Table 1 (Eurofins), and quantification was performed using the comparative CT method (2^(-ΔΔCt)) with *Rplp0* as the housekeeping gene.

**Table 1.** Primer used for the realtime-PCR.

Gene	Forward (5’-3’)	Reverse (5’-3’)
*Tfeb*	TGTCTAGCAGCCACCTGAAC	TGTCTAGCAGCCACCTGAAC
*Ppara*	GTGGTGCATTTGGGCGTATC	TGAACTTCAACTTGGCTCTCCT
*Lamp1*	GCCCTGGAATTGCAGTTTGG	TGCTGAATGTGGGCACTAGG
*Rplp0*	GGCGACCTGGAAGTCCAACT	CCATCAGCACCACAGCCTTC

### Immunohistochemistry

Brains were collected, immersion-fixed in 4% paraformaldehyde for 24 h and cryopreserved in 30% (w/v) sucrose solution. Coronal sections (35 μm) were cut using a freezing microtome and stored at −20°C in 50 mM phosphate buffer (pH 7.4) containing 30% glycerol and 30% ethylene glycol. For the fluorescent labeling of tdTomato in dopaminergic neurons, free-floating tissue sections were incubated with 5% normal goat serum (NGS; Thermo Fisher Scientific 31,873) in 1X Tris-buffered saline (TBS; Thermo Fisher Scientific, J62662.K3; pH 7.6) containing 0.25% Triton X-100 (Merk, 9036-19-5; TBS-T) for 1 h at room temperature in order to block nonspecific binding sites. Mouse endogenous IgGs were blocked with excess (40 μg/ml) unconjugated Fab fragments in TBS-T (Jackson Immunoresearch, 115-007-003) for 1.5 h at room temperature. Sections were incubated with the primary rabbit anti-red fluorescent protein (RFP) antibody (1:3000; Rockland, 600-401-379) for 48 h in 1% bovine serum albumin (BSA; Merk, A8022) in TBS-T at 4°C. tdTomato labeling was achieved by incubating the secondary anti-rabbit IgG (H+L)-DyLight 594 antibody (1:300; Vector Laboratories, DI-1594-1.5) for 1 h at room temperature. Tissue sections were washed and dopaminergic neurons were stained with directly conjugated anti-TH antibody, clone LNC1, Alexa Fluor 488 Conjugate (1:100; Sigma-Aldrich, MAB318-AF488,) for 3,5 h at room temperature. Sections were then washed, mounted on coated slides and coverslipped with an aqueous-based fluorescence antifade mounting medium. For the fluorescent labeling of tdTomato in cholinergic neurons, tissue sections were incubated with 5% normal donkey serum (Sigma-Aldrich, S30-M) in TBS-T for 1 h at room temperature and incubated with the following primary antibodies: rabbit anti-RFP antibody (1:3000, Rockland, 600-401-379) for 48 h and goat anti-CHAT antibody (1:200, Sigma-Aldrich, AB144P,) for 24 h, both in 1% BSA in TBS-T at 4°C. Labeling was achieved by incubating the following secondary antibodies: anti-rabbit IgG (H+L)-DyLight 594 (1:300; Vector Laboratories, DI-1094,) and anti-goat IgG (H+L)-Alexa Fluor 488 (1:500; Abcam, ab150129). Sections were then washed, mounted on coated slides and coverslipped with an aqueous-based fluorescence antifade mounting medium. For brightfield microscopy, free-floating brain sections were quenched by incubation in a mixture of 3% H_2_O_2_ and 10% methanol in TBS. Nonspecific binding sites were blocked using 5% NGS in TBS-T. Samples were incubated in a TBS-T solution containing 1% BSA and rabbit anti-RFP (1:20000; Rockland, 600-401-379) for two days at 4°C. Sections were rinsed and incubated in a biotinylated secondary antibody solution (goat anti-rabbit, 1:200, Vector Laboratories, BA1000,) with 1% BSA in TBS-T. Following treatment with avidin-biotin-peroxidase (Vector Laboratories, ABC-HRP kit; PK-6100), color reaction was developed using 3,3’-diaminobenzidine kit (Vector Laboratories, SK-4100). Sections were mounted on coated slides, coverslipped with Depex (Sigma-Aldrich 06,522). Images of the tissues were acquired using a Zeiss Observer.Z1 microscope (Carl Zeiss) with a 20× and 63× objective. Representative images and panels were generated in Fiji and Illustrator (Adobe).

### Statistics

Data are presented as the mean with standard deviation unless otherwise specified in the figure legend. Statistical analyses were conducted using Prism 7 (Version 8.00, GraphPad Software Inc.). t-tests were utilized to determine significant differences in means between two groups. One-way ANOVA was employed to assess significant differences in means among three or more independent groups, followed by post hoc Tukey’s test for comparing every mean with every other mean, or Dunnett’s test to compare each mean with a control mean. Two-way ANOVA, followed by Sidak’s post hoc test, was used to assess the impact of two factors on the responses in a multiple comparison. A p-value less than 0.05 was considered statistically significant.

## Supplementary Material

Supplementary table S1toS4 R4.xlsx

Supplementary Material R4.docx
