# Supplementary material for: Design and validation of a reporter mouse to study the dynamic regulation of TFEB and TFE3 activity through in vivo imaging techniques
Source: Autophagy. 2024 Mar 24;20(8):1879–94. doi: 10.1080/15548627.2024.2334111 (PMC11262230; doi:10.1080/15548627.2024.2334111)
Supplement: Supplementary Material R4.docx [file KAUP_A_2334111_SM0713.docx]

**Title**

Design and validation of a reporter mouse to study the dynamic regulation of TFEB and TFE3 activity through *in vivo* imaging techniques

**Authors**

Electra Brunialti^a^, Nicoletta Rizzi^b^, Rita Pinto-Costa ^c^, Alessandro Villa^a^, Alessia Panzeri^a^, Clara Meda^a^, Monica Rebecchi^a^, Donato A. Di Monte^c^, Paolo Ciana^a^

**Affiliations**

^a^ Department of Health Sciences, University of Milan, Milan, Italy;

^b^Animal Care Unit, University of Milan, Milan, Italy;

^c^ German Center for Neurodegenerative Diseases (DZNE), Bonn, Germany

**This file includes:**

Supplementary material and methods

Supplementary figures 1 – 5

**Supplementary Materials and Methods**

***Protein isolation and western blotting***

Mouse lungs derived from vehicle or torin1-treated mice were homogenized at 4°C in RIPA lysis buffer (50 mM Tris-HCl, pH 7.4, 150 mM NaCl, 1% NP-40 [Thermo Fisher Scientific, 85124], 0.5% sodium deoxycholate [Thermo Fisher Scientific, J62288.30], 0.1% sodium dodecyl sulfate [SDS], protease, and phosphatase inhibitors [Thermo Fisher Scientific, A32955; A32957]) using mechanical dissociation. Protein concentration was determined using the standard Bradford method (Thermo Fisher Scientific, 23200). SDS and β-mercaptoethanol were added to samples before boiling, and equal amounts of proteins (40 μg/lane) were separated by 4-12% SDS-polyacrylamide gel electrophoresis. Proteins were then transferred onto a nitrocellulose membrane. The membrane was probed using anti-phospho-RPS6/S6 ribosomal protein (Ser 235/236; Cell Signaling Technology, 4856). After incubation with the appropriate horseradish peroxidase (HRP)-conjugated secondary antibody (Vector, PI1000), bands were visualized using the LiteAblot Extend Long Lasting chemiluminescent substrate (Euroclone, EMP013001) with the Odyssey (Li-Cor) imaging system. To monitor potential artifacts in loading and transfer among samples in different lanes, the blots were rather treated with StripAblot Stripping Buffer (Euroclone, EMP100500) and re-probed with monoclonal antibody to RPS6 ribosomal protein (Cell Signaling Technology, 2217). Bands were quantified for densitometry using ImageJ software.

**
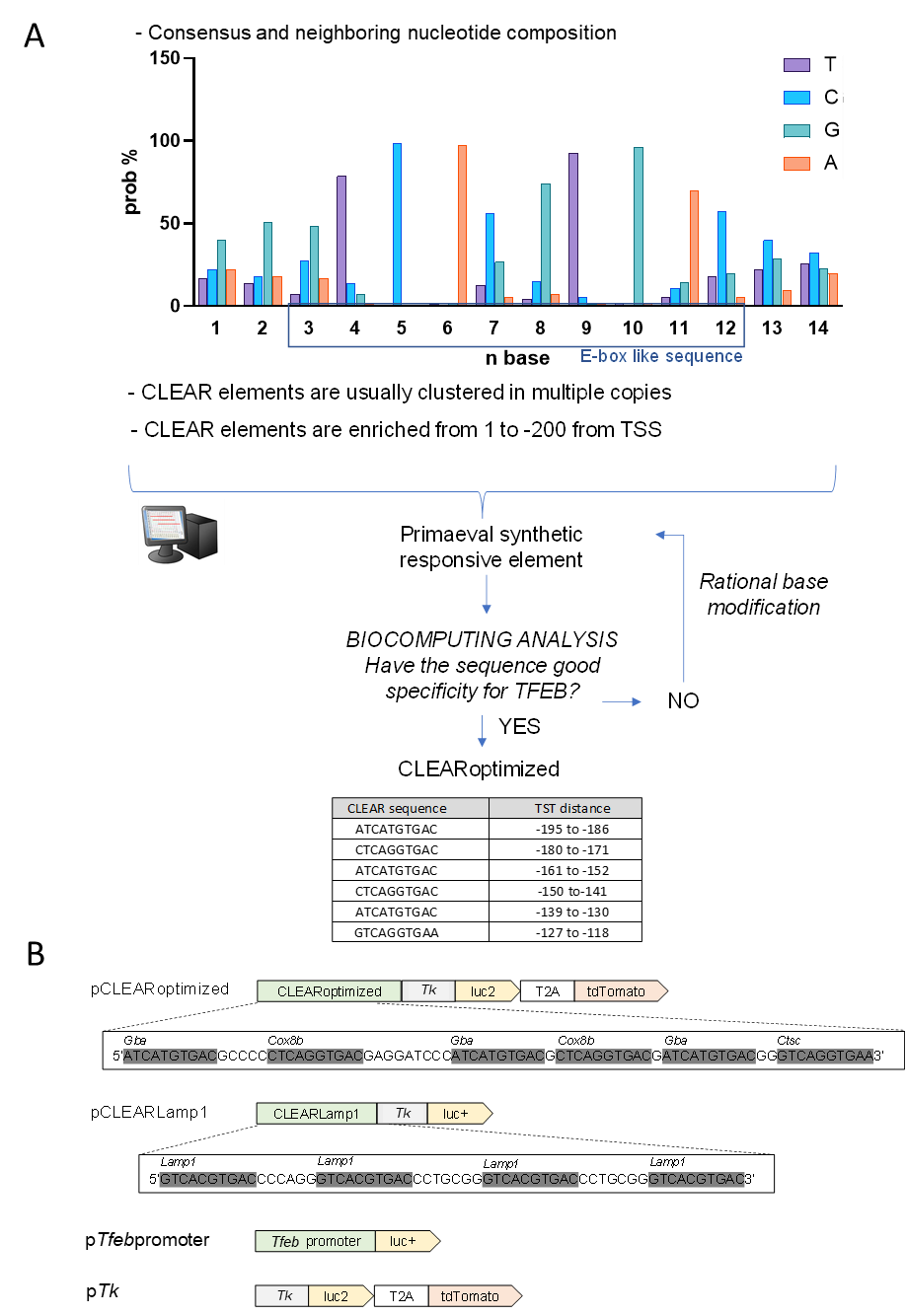
**

**Figure S1.** Reporter vector design and description. (**A**) bioinformatics analysis of the promoter regions of 128 TFEB-target genes and scheme of iterative process to generated CLEARoptimized sequence**. (B**) Schematic representation of the reporter vectors: the sequences of the responsive elements are shown for pCLEARoptimized and pCLEARLamp1, with the CLEAR sequences highlighted (genes where they belong are also indicated in the upper part of the responsive element). CLEARoptimized: synthetic TFEB responsive element developed in this study; CLEARLamp1: CLEAR elements from the *Lamp1* gene; *Tfeb* promoter: 2000 bp of the *Tfeb* promoter; *Tk*: minimal thymidine kinase promoter; luc2: optimized firefly luciferase 2; luc^+^: optimized firefly luciferase; T2A: self-proteolytic peptide; tdTomato: tdTomato red fluorescent protein. The plasmid p*Tk*, which does not contain responsive elements, was used as a negative control.

**Figure S2.** Validation of pCLEARoptimized. (**A**) Luciferase activity was measured in RCS cell line transiently transfected with pCLEARoptimized and grown in complete (Fed) media, at 24 h the media was replaced with fresh complete media. Data represent FC of normalized RLU versus vehicle ± SD (n = 3) in duplicate; no statistical significance identified *versus* time0 with one-way ANOVA followed by Dunnett’s multiple comparisons test. (**B**) Luciferase activity was measured in HeLa cell line transiently transfected with pCLEARoptimized and grown in complete (Fed) or diluted (Starved) media. At 24 h, the media was replaced with complete media in both conditions (re-fed). Data represent FC of normalized RLU versus vehicle ± SD (n = 3) in duplicate; ***p< 0.0001 versus time 0 calculated with one-way ANOVA followed by Dunnett’s multiple comparisons test. (**C**) Luciferase activity was measured in HeLa cell line transiently transfected with the pCLEARoptimized and treated with agents capable of eliciting TFEB activation for 16 h: chloroquine (chloroq.), trehalose (treh.), ambroxol (ambr.). Bars represent fold change (FC) of normalized RLU versus vehicle ± SD (n = 3) in duplicate; **p < 0.005, ***p< 0.0001 versus vehicle calculated with one-way ANOVA followed by Dunnett’s multiple comparisons test. **(D)** Torin 1 is able to elicit fast TFEB nuclear translocation. Representative time-lapse pictures of HeLa cells transiently transfected with the pCMV-TFEB-GFP vector and treated with 2 µM of torin 1 or vehicle. Image taken at time 0 and 2 h after the treatment, in white the fluorescence emitted from the TFEB-GFP fusion protein. Scale bars: 20 μm.

**Figure S3.** TFEB-STOP and TFEB-*luc2* luciferase expression. (**A**) Screening of the clone containing the entire transgene. Six different diagnostic PCR were performed on selected clones to confirm the correct insertion of the full transgene in chromosome 1. The clone was used to generate TFEB-STOP mice. (**B**) Representative image of the bioluminescence emitted *in vivo* and in dissected organs in TFEB-STOP mouse. Scale bars: 1 cm. (**C**) Bioluminescence quantification of the whole body of TFEB-STOP and TFEB-*luc2* mice. Data are reported as total flux (p/s), (n=3), statistical significance calculated with an unpaired *t*-test, ****p*<0.001. (**D**) The *in vivo* bioluminescence emitted from the head, thorax, abdomen, and pelvic region was quantified in four mice (2 male and 2 female) on days 104 and 123 after the birth. Left: Representative image of the bioluminescence emitted *in vivo*. Right: data are reported as bioluminescence signal (p/s/cm^2^/sr) normalized on day 104.


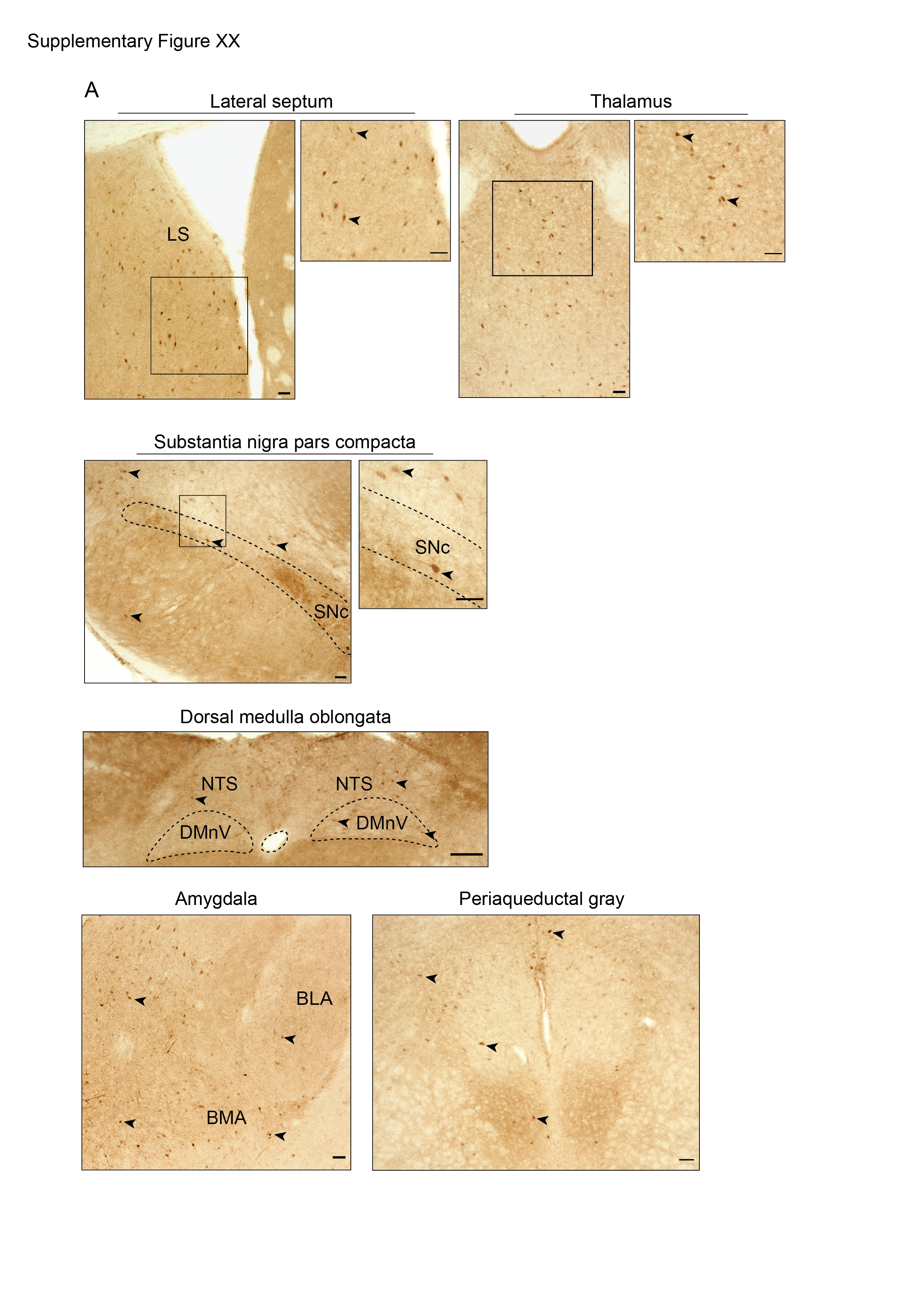


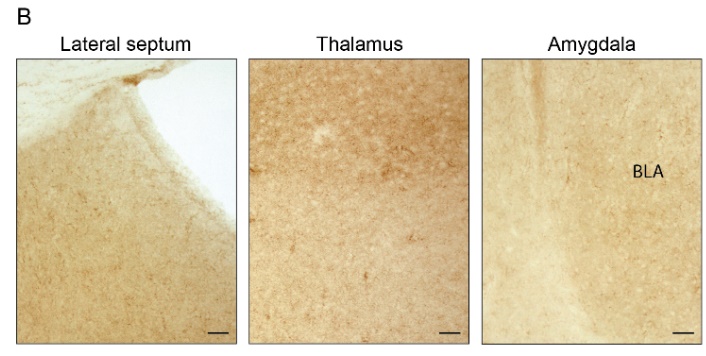


**Figure S4.** tdTomato expression in TFEB-luc2 and wild-type (control) mice. (**A**) Representative brightfield images of tdTomato expression in coronal sections of different brain regions of TFEB-luc2 mice. Arrowheads indicate some of the tdTomato-immunoreactive cells. The substantia nigra pars compacta and dorsal motor nucleus of the vagus nerve are delineated by dashed lines. Scale bars: 50 μm. (**B**) Representative brightfield images of tdTomato immunostaining in coronal sections of different brain regions of wild-type mice. Scale bars: 50 μm. BLA: basolateral amygdala; BMA: basomedial amygdala; DMnV: dorsal motor nucleus of the vagus nerve; LS: lateral septum; NTS: nucleus of the solitary tract; SNc: substantia nigra, compact part.

**Figure S5.** *In vivo*-jetPEI delivery ensures heterologous expression of luciferase and TFEB in the lung 24 h after injection. (**A**) Representative time course of the in vivo bioluminescence emitted from a wild type mouse intravenously injected with pCMV-*luc2* plasmid encapsulated in In vivo-jetPEI, 0, 16, 24 h after the plasmid injection, and the organs explanted at the end of the experiment (24 h). Pseudocolor images of mice were obtained 15 min after the injection of 80 mg/kg of luciferin with 5 min exposition time and reported as the corresponding scale bar. (**B)** Total RNA was purified from the dissected lungs of mice injected with pCMV-TFEB or an empty vector, and the expression of *TFEB* and housekeeping genes (*ActB* and *Rplp0*) was analyzed using real-time PCR. Relative quantification of the transcripts was obtained using the 2^-ΔΔCt^ method. (**C**) Western blot analysis of lungs homogenate derived from vehicle or torin1-treated mice, showing phosphorylated RPS6 at Ser235/236 (p-RPS6) and total RPS6 as internal standard; Right panels: densitometric analysis of phosphorylation levels. Statistical significance *p < 0.05, vs Vehicle calculated by t-test (n=2). (**D**) Bioluminescence signals were acquired for each organ obtained from mice subjected to 48 h of starvation or normal feeding and the measurements of bioluminescence signal are presented in the graph as fold change (FC) of the radiance photons of starved versus fed animals and presented as mean ± SD. Statistical significance was determined by multiple t-test versus fed animals. *p < 0.05, **p < 0.01. Showing that an increase in luciferase activity is detectable in both bone and bone marrow.
